# Diabetes educator role boundaries in Australia: a documentary analysis

**DOI:** 10.1186/s13047-017-0210-9

**Published:** 2017-07-10

**Authors:** Olivia King, Susan Nancarrow, Sandra Grace, Alan Borthwick

**Affiliations:** 1 School of Health and Human Sciences, Southern Cross University, Military Road, East Lismore, Sydney, 2480 Australia; 2 Centre for Innovation and Leadership in Health Sciences, Faculty of Health Sciences, University of Southampton, East Lismore, SO17 1BJ England

**Keywords:** Diabetes educator, Diabetes education, Interdisciplinary, Interprofessional, Role boundaries

## Abstract

**Background:**

Diabetes educators provide self-management education for people living with diabetes to promote optimal health and wellbeing. Their national association is the Australian Diabetes Educators Association (ADEA), established in 1981. In Australia the diabetes educator workforce is a diverse, interdisciplinary entity, with nurses, podiatrists, dietitians and several other health professional groups recognised by ADEA as providers of diabetes education. Historically nurses have filled the diabetes educator role and anecdotally, nurses are perceived to have wider scope of practice when undertaking the diabetes educator role than the other professions eligible to practise diabetes education. The nature of the interprofessional role boundaries and differing scopes of practice of diabetes educators of various primary disciplines are poorly understood. Informed by a documentary analysis, this historical review explores the interprofessional evolution of the diabetes educator workforce in Australia and describes the major drivers shaping the role boundaries of diabetes educators from 1981 until 2017.

**Methods:**

This documentary analysis was undertaken in the form of a literature review. STARLITE framework guided the searches for grey and peer reviewed literature. A timeline featuring the key events and changes in the diabetes educator workforce was developed. The timeline was analysed and emerging themes were identified as the major drivers of change within this faction of the health workforce.

**Results:**

This historical review illustrates that there have been drivers at the macro, meso and micro levels which reflect and are reflected by the interprofessional role boundaries in the diabetes educator workforce. The most influential drivers of the interprofessional evolution of the diabetes educator workforce occurred at the macro level and can be broadly categorised according to three major influences: the advent of non-medical prescribing; the expansion of the Medicare Benefits Schedule to include rebates for allied health services; and the competency movement.

**Conclusion:**

This analysis illustrates the gradual movement of the diabetes educator workforce from a nursing dominant entity, with an emphasis on interprofessional role boundaries, to an interdisciplinary body, in which role flexibility is encouraged. There is however, recent evidence of role boundary delineation at the meso and micro levels.

**Electronic supplementary material:**

The online version of this article (doi:10.1186/s13047-017-0210-9) contains supplementary material, which is available to authorized users.

## Background

In Australia health care services and policy makers face significant challenges as they endeavour to meet population health needs in the current climate, characterised by economic uncertainty, enduring workforce shortages, an ageing population and burgeoning rates of chronic disease [1–4]. A significant proportion of the health care budget is consumed by the workforce, prompting more innovative approaches to the delivery of health care [1–4]. Contemporary service designs featuring interdisciplinary collaboration and role flexibility have been suggested to enhance efficiency, effectiveness and economy in health care delivery [1, 3–5]. Genuine role flexibility may see health professionals undertake clinical tasks that were traditionally considered the purview of another health profession, with an emphasis on meeting service-user needs [1, 4, 6, 7]. This contemporary approach to health care delivery challenges many of the customs embedded in health care practice and can lead to interprofessional contestation at the macro, meso and micro levels, as the professions renegotiate their roles boundaries [1, 8, 9]. Numerous studies have explored inter- and intra-professional role boundary negotiations in the context of modernizing changes to health systems and services, many of which focused on the micro-level negotiations [5, 10, 11].

Diabetes is a chronic health condition and is considered to be the epidemic of the twenty-first century with the prevalence increasing exponentially [12]. Diabetes educators are among the range of health care providers working to reduce the impact of diabetes at an individual and population level. The Australian Diabetes Educators Association (ADEA), established in 1981, is the national association. ADEA recognises registered nurses (RN), podiatrists, accredited practising dietitians (APD), pharmacists and several other health professional groups as providers of diabetes education and eligible to become a credentialled diabetes educator (CDE) [13]. The nature of diabetes education lends this clinical area to role flexibility however, as this paper illustrates, in Australia the flexibility of this faction of the workforce has been limited.

In Australia nurses have historically filled the diabetes educator role and anecdotally nurses are perceived to have a wider scope of practice when working in the diabetes educator role than those of the other CDE eligible professions. The nature of the perceived differences in the roles and scopes of practice of diabetes educators of varying primary disciplines in Australia is unclear. Internationally there are different systems and processes governing diabetes education practices, both historically and currently. With such variability it was determined this analysis would focus only on the Australian context.

The diabetes educator workforce has existed in a social climate influenced by economic uncertainty, an ageing population, increasing rates of chronic disease, health workforce shortages, legislative changes and other factors. This paper explores the macro, meso and micro level factors which have both shaped and have been indicative of change within the diabetes educator workforce in Australia from 1981 to 2017. For this review, macro level events are defined as those occurring at a national or government level, which impact the health workforce more widely. Meso level events are defined as those occurring at the professional association level, affecting some or all members of the diabetes educator workforce. Micro-level events are defined as those occurring at a local or workplace level and affect specific individuals or groups of diabetes educators.

## Methods

A documentary analysis was undertaken, structured as a literature review. It retained several principles of a systematic review, including the identification of search terms, search engines, specified inclusion and exclusion criteria and other limitations; however not all of the attributes of a systematic review were included. On planning the literature search, a decision was made to include both peer-reviewed research and grey literature. Grey literature is produced by a range of entities, including governments and government agencies, academic institutions and professional associations. Grey literature may be available in printed and electronic formats and, given that it is not published commercially, this type of literature is generally publically accessible [14].

Benzies, Premji, Hayden and Serrett [14] discussed the rationale for including grey literature in state-of-evidence reviews. The authors described six indicators to guide the researcher in their consideration of grey literature, of which two indicators relate to the availability of sufficient and high quality evidence and one to a general consensus around the evidence. An initial search of peer reviewed literature relating to diabetes educator role boundaries illustrated a paucity of evidence. Consequently, the inclusion of grey literature was not only beneficial for this analysis, but was necessary to complete it.

Since its establishment in 1981, ADEA has published a number of documents which, at the time of their publication, related to and reflected the role and scope of practice of diabetes educators. These publications were developed by ADEA to provide guidance and reference to its membership. There has been a number of other documents, predominantly grey literature, which relate to the diabetes educator role and scope of practice in the Australian context, including government publications, government agency reports, gazettes and legislation. The mnemonic STARLITE (sampling strategy, type of literature, approaches, range of years, limits, inclusions and exclusions, terms used, electronic sources) was used to develop a systemic framework to guide the retrieval of literature [15]. A STARLITE framework guided the process of documentary data retrieval, which began with a comprehensive search of the ADEA website, followed by a citation search (Table 1). Peer-reviewed literature has also been consulted for this analysis. A separate STARLITE framework guided a database literature search (Table 2). In cases where details of relevance were not contained within any retrieved documents, targeted personal communication was utilised to gather the information. Individuals who were deemed to be credible and who had access to records which contained these details were contacted to supply or confirm specific details.

**Table 1:** STARLITE Framework 1

Sampling strategy	Purposive
Type of literature	Grey literature; ADEA publications
Approaches	Grey literature search, comprehensive ADEA website search, citation search, consultation with key experts
Range of years	−1981 – Current (June 2017)
Limits	English, human
Inclusions and exclusions	Included: ADEA guidelines, position statements, policies, submissions, codes of conduct, standards of practice, role and scope of practice documents, Annual Reports, AGM minutes, Board updates, scoping documents, research papers, newsletters, member news, information sheets, project information Excluded: Consumer resources, order forms, promotional materials, product information and advertising guidelines, business and private practice resources, frequently asked questions
Terms used	Not applicable: the website was searched comprehensively
Electronic resources	ADEA website

**Table 2:** STARLITE Framework 2

Sampling strategy	Purposive sampling; search for documents relating to the role and scope of practice of diabetes educators in Australia
Type of literature	Peer reviewed literature and grey literature
Approaches	Subject search, citation search, grey literature search
Range of years	1981 – Current (June 2017)
Limits	English, human, Australia
Inclusions and exclusions	Inclusions: Australian diabetes educators or diabetes education; diabetes educator role and scope of practice; published opinion pieces Exclusions: International documents; documents with no direct relevance to the Australian diabetes educator role and scope of practice
Terms used	Diabetes education, diabetes educator
Electronic resources	CINAHL (EBSCO) and Medline Plus (EBSCO)

Most documents included in this review were grey literature. Therefore, the application of standard assessment tools to determine the quality of the literature retrieved was not indicated. Decisions relating to the quality of the documents retrieved were made with regards to Scott’s [16] criteria to evaluate the legitimacy of documentary evidence: authenticity, credibility, representativeness and meaning.

Synthesis and Analysis In synthesising and analysing the findings from the documents retrieved, key drivers were identified as events or influences that appeared to shape the role boundaries of diabetes educators. These drivers were categorised as macro, meso or micro level and charted on a table in chronological order, in effect creating a timeline. The timeline was analysed and the drivers were further categorised according to emerging themes. Key themes were identified as pivotal historical events or movements which appeared to precede changes in the wording within documents published subsequently by ADEA, which were indicative of an evolving interdisciplinary culture.

### Search results

**Fig. 1:**
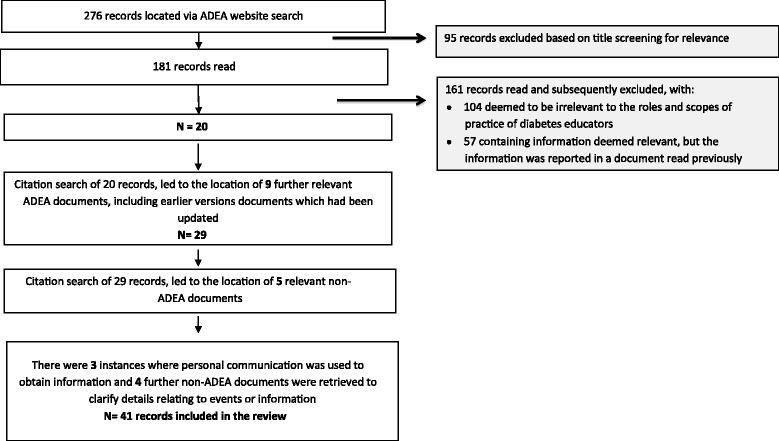
PRISMA diagram (ADEA website and grey literature search)

**Fig. 2:**
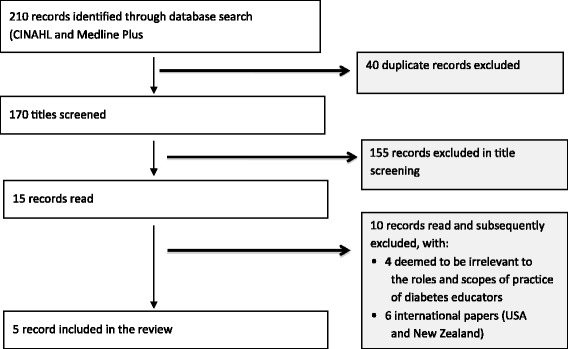
PRISMA Diagram (Database Search)

The ADEA website search yielded 276 records and the database searches yielded 210 records. A total of 46 records were included in the documentary analysis. Figures 1 and 2 illustrate the processes used to exclude irrelevant records and to locate further relevant ones to enable the gathering of sufficient data for review. Documents retrieved via the ADEA website and database search were included if they related to the role and scope of practice of diabetes educators in Australia. International documents were excluded as the systems and practice of diabetes education in other countries vary. Any advances in diabetes education practice and the roles and scopes of practice of diabetes educators in other countries may not be relevant to the evolution of diabetes education and the influences on the professional role boundaries of diabetes educators in Australia.

Each of the written documents cited in this paper appear in the reference list. The details of all documents contributing to the analysis are contained within Additional file 1.

### Quality indicators

Scott’s [16] four criteria were used to assess the legitimacy of documents retrieved in this analysis. The first criterion is authenticity or the degree to which documents can be considered genuine. The second is the credibility or trustworthiness of the source of the document. The third is the degree to which the document is representative of the phenomenon of interest. According to Scott [16] representativeness can be evaluated according to two sub-categories: survival and availability. Survival and availability refer to the extent to which the document is stored in a safe and accessible place, such as a public library. The final criterion is meaning, which is considered more difficult to evaluate. It requires the researcher to interpret the intended meaning of the document, by understanding the circumstances in which the document was produced.

The 46 documents were evaluated against these four criteria, with each document deemed to meet the criteria and therefore included in the review. Additional file 2 provides an overview of the types of documents included in this review, including the source. It also demonstrates the application of Scott’s [16] criteria used to evaluate the legitimacy of the 46 documents included.

## Results

The documents included in the review provided sufficient information to trace the interprofessional evolution of the diabetes educator role from the inauguration of ADEA until June 2017. The evolution is described according to four drivers which became evident throughout the analysis. These drivers are presented as sub-sections and will be prefaced by an overview of the evolution of the diabetes workforce in adopting an increasingly interprofessional profile and function.

### The evolution of the diabetes workforce: Moving towards interprofessional roles

ADEA was established in 1981. In a paper published in 1984, *Diabetes education in Australia,* the author and first ADEA president described the unstructured manner in which diabetes education was provided by designated nurses in the hospital setting in 1970s and the circumstances that led to the inauguration of ADEA. The first outpatient Diabetes Education Centre in Australia was established at the Royal Newcastle Hospital in 1974. In the decade that followed, there was significant growth in the clinical area of diabetes education and it became recognised as a health care specialty [17]. Although the author acknowledged the tendency for diabetes education to be provided using a team-based approach, role boundaries in diabetes education were delineated, ‘All newly diagnosed diabetics both type I and type II received guidance with their individual meal plans from the dietitians and diabetes education from the nurse educator’ ([17], p. 22). ADEA’s membership which was 300 strong, included nurses, podiatrists, dietitians, medical officers, psychologists, pharmacists, occupational therapists and ‘lay diabetes educators’ ([17], p. 23).

In 1986, ADEA introduced the certification trademark Credentialled Diabetes Educator (CDE) [18]. In 1989, *The Role Statement of the Diabetes Nurse Educator* was published [19, 20], which suggested diabetes education was considered part of the nursing remit. In 1991, *National Standards of Practice for Diabetes Educators* was published. It referred to the ‘multiplicity of professional backgrounds and experiences of diabetes educators’ ([21], p. 1) however it did not specify those health professions eligible for ADEA credentialling.

In 1994, *National Guidelines for the Safe Practice of Diabetes Nurse Educators* was published. As the title suggests, this document related specifically to nurse diabetes educators and discussed the ethico-legal dilemmas that may be encountered in practice. While the document stated, ‘diabetes management is increasingly considered to be team care’ ([22], p. 4), no other non-medical health professions were mentioned.

In 1996, *National Core Competencies for Diabetes Educators* was published. At that time, ADEA recognised nurses, dietitians, podiatrists, psychologists and social workers as the professions providing specialised care for people with diabetes. This document referred members to the legal, ethical and professional standards of their primary disciplines to guide diabetes education practice [23]. The document detailed five units of competency. Unit 1.4 described a competent diabetes educator as one that, ‘Maintains and applies clinical skills appropriate to the educator's clinical discipline and their specialist function, for example, nurses: insulin dosage adjustment or correct injection technique, dietitians: diabetes dietary prescription, podiatrists: wound management’ ([23], p.3). The role boundaries between the different primary professions working in the diabetes education realm were delineated here.

In the 1990s, the Dietitians Association of Australia approached ADEA for approval for eligibility for credentialling. Approval was granted and in 1999 the first dietitian successfully became a CDE (Per Communication, 30th August, 2016). In 2001 *Role of the Diabetes Educator in Australia* was updated. The revised document acknowledged that since the publication of the first role statement for nurse diabetes educators in 1989, there had been a growth in the size and diversity of the ADEA membership [19].

In 2001, *Credentialling of Diabetes Educators 2000* was published. It stated that diabetes educators must have a base qualification as either RN, dietitian, podiatrist, psychologist, medical officer or Aboriginal health worker. In 2003, the *National Standards of Practice for Diabetes Educators* document was updated. This document listed the professions able to practise diabetes education as nursing, dietetics, podiatry, psychology, medicine, physiotherapy and Aboriginal health workers [24]. In 2005 a joint statement between the Dietitians Association of Australia (DAA) and ADEA was published. This document indicated that at the time, role overlap in diabetes education had increased: ‘ ... opportunities for expanded spheres of practice have resulted in the practice of diabetes education becoming more *interdisciplinary* in nature’([25], p.1). As such, the roles of the dietitian and diabetes educator required clarification.

The first podiatrist achieved CDE status in 2004 (Per Communication, 3rd October, 2016). In 2005, *All about diabetes educators – a guide for General Practitioners*, was published. This article stated that only RN CDEs were qualified to sign National Diabetes Services Scheme forms, confirming a person’s diagnosis of diabetes [26]. In 2007 registered pharmacists accredited to conduct medication management reviews were deemed eligible to achieve CDE status [27]. In 2007 *The Credentialled Diabetes Educator in Australia – Role and Scope of Practice* was updated. It stated, ‘In light of the expanding role of Credentialled Diabetes Educators, the ADEA completed a review of the health disciplines that it recognises as eligible for credentialling in 2007’ [20, p.7]. The findings of the review were that RNs, dietitians, registered pharmacists (accredited to conduct medication management reviews) and medical officers were CDE eligible. Podiatrists, who had previously been approved CDE eligible, were not listed. Consistent with the previous role and scope of practice documents, the 2007 version stipulated that each CDE’s role, scope of practice and provision of clinical care is congruent with that of their primary profession.

In 2008, registered podiatrists were deemed CDE eligible [28]. The first pharmacist achieved CDE status in 2009 (Per Communication 30th August, 2016). In 2012, accredited exercise physiologists became CDE eligible [29]. In 2014, an application was made to ADEA to repeal the requirement for pharmacists to have medication management accreditation in order to be considered eligible for CDE status [18].

In 2015, direct entry midwives and physiotherapists were approved as CDE eligible [18]. Also in 2015, the joint position statement between the Dietitians Association of Australia and ADEA was updated. This was the earliest document included in this review that emphasised the interdisciplinary nature of the diabetes educator role and diabetes self-management education (DSME):

Regardless of primary health discipline background, all CDEs are eligible to undertake all aspects of DSME. The extent of DSME provided by a CDE does not depend on their primary health discipline but is dependent on individual self-determined role and scope of practice ([30], p.8).

It stated that all CDEs are qualified to sign NDSS forms and eligible to claim Medicare, DVA and private health insurance rebates where applicable, for DSME services.

In 2015, the *Role and Scope of Practice for Credentialled Diabetes Educators in Australia* was updated. Like the preceding version, this document emphasised that the role and scope of practice of a CDE is influenced by factors including legislation, professional experience, training, competency, workplace policies and others. Unlike preceding versions, which referred to discipline-dependent scope of practice, this document referred to ‘individual scope of practice’ ([13], p.14).

In 2016, there had been a notable increase in CDEs of dietetics and podiatry background in the preceding year [31]. A Communiqué was sent to all ADEA members entitled, *Working for All Members*. It stated:

ADEA values and supports all its members and does not privilege or promote one discipline over another ... In creating messaging to government National Office and the Board seek expert advice. It is important that feedback is brief and very targeted. Trying to sell different versions of CDE weakens the message significantly, creates confusion and dramatically reduces interest in the topic as the key message is lost ... As you would be aware, especially in a political reality where there are continued major cuts to health care funding, any perception of division within a representative organisation is likely to result in ADEA’s issue not being prioritised. Division adversely impacts the authority of the organisations’ standing with the relevant government department and can undermine the arguments for change [32].

This was a significant and overt action by ADEA to reduce perceived interprofessional role boundaries within the membership. While this communiqué demonstrated that the ADEA promoted an inclusive, interdisciplinary culture within the diabetes educator workforce, it exemplified the perception of enduring interprofessional boundaries. The communiqué discussed ADEA’s work advocating that CDEs of all disciplines have the right to authorise patient access to additional blood glucose test strips (BGTS) via the National Diabetes Services Scheme. ADEA was successful in this endeavour and stated that, ‘If the ADEA’s position was that only nurse CDEs should be able to authorise BGTSs, then ADEA would have been at risk of losing credibility and not being heard.’ [32]. This indicates that, at the micro level at least, there were enduring perceptions of interprofessional role boundaries in the diabetes educator world and ongoing attempts to protect task domains.

### The non-medical prescribing era

Insulin is one of the main medications used to manage diabetes. In 1994 ADEA published *National Guidelines for the Safe Practice of Diabetes Nurse Educators*. This document addressed several ethico-legal considerations for nurses providing diabetes education and stated that diabetes nurse educators were, ‘... responsible for teaching the patient insulin technique including appropriate insulin adjustment. It is important for individual educators to clarify, and have documented practice guidelines, with respect to medication adjustment, with their employing body’ ([22], p.5). At this time, insulin was a schedule III drug, which meant it could be purchased from a pharmacy without a prescription. This document further stated, ‘nurses cannot prescribe insulin. Therefore any medication adjustment must occur under the standing orders of the doctor’ ([22], p.10).

The *National Core Competencies for Diabetes Educators* was published in 1996. It provided examples of clinical tasks undertaken by diabetes educators specific to their primary discipline. Unit 1.4 stated that a diabetes educator, ‘Maintains and applies clinical skills appropriate to the educator's clinical discipline and their specialist function, for example, nurses: insulin dosage adjustment or correct injection technique ... ’ ([23], p.3). Insulin adjustment was considered part of the nurse diabetes educator’s role at that time.

In March 2000, insulin was rescheduled from schedule III to a schedule IV drug. Consequently, as of December 2000 only a medical practitioner could prescribe insulin [33, 34]. This legislative change meant RN diabetes educators’ autonomy was diminished significantly. Subsequently, a group of RN CDEs in New South Wales successfully lobbied for the right to issue a seven day supply of insulin to patients in accordance with a prescription from a medical practitioner [34, 35]. This delineated the boundaries between RN and non-nurse diabetes educators, in New South Wales at least.

The *National Core Competencies for Diabetes Educators* was updated in 2001. Like the 1996 version, five units of competency were defined, unit 1.4 providing examples of discipline-specific diabetes educator practices, ‘nurses: insulin dosage adjustment or correct injection technique ... ’ ([36], p.3). This indicates that in 2001, insulin adjustment was considered part of the nurse diabetes educator role, despite insulin being rescheduled to a prescription-only medication the previous year.

In 2004, ADEA published *National Standards for the Development and Quality Assessment of Services Initiating Insulin Therapy in the Ambulatory Setting* which outlined a number of standards. Structure Standard 2.1 stated, ‘Registered Nurse Diabetes Educators and Dietitian Diabetes Educators who undertake a coordinating and primary role in the ambulatory initiation of insulin therapy have a minimum of 12 months supervised, relevant clinical experience’ ([37], p.23).

In 2007, *The Credentialled Diabetes Educator in Australia: Role and Scope of Practice* was published. It stated that some CDEs have a role in ‘specific aspects of diabetes care, such as insulin initiation and stabilisation’ ([20], p.11). There was an apparent decline in the emphasis on the RN CDE’s role in insulin adjustment. In 2008, the *National Core Competencies for Diabetes Educators* was updated. This version omitted references to specific clinical applications such as insulin adjustment [38].

In June 2009, legislation was passed enabling podiatrists in Victoria, with relevant endorsement, to prescribe schedule IV drugs according to a formulary [39]. In 2010, an article was published in the Diabetes Management Journal, *Nursing roles in initiating and adjusting insulin.* The author and past ADEA president discussed circumstances in which RNs were able to prescribe insulin: with endorsement as a nurse practitioner (NP) or with a service protocol [40].

In 2012, there was a resolution passed at the ADEA annual general meeting (AGM) whereby several RN ADEA members requested the ADEA Board lobby the federal government to secure secondary prescribing rights for RN CDEs, [41]. In 2013, ADEA made a submission to the Nursing and Midwifery Board of Australia entitled *Proposed expanded endorsement for scheduled medicines. Draft Registration standard for endorsement of registered nurses and/or registered midwives to supply and administer scheduled medicines under protocol* *.* As the title suggests, this document sought to advance nursing’s bid to secure insulin prescribing rights for RN CDEs [42]. Subsequently, ADEA informed members of their progress in a communiqué, ‘In summary, the ADEA Board will continue to take every opportunity to advocate for the recognition of RN CDEs to have secondary prescribing rights (regarding the adjustment of insulin therapies) ... ’ [34].

In 2014, ADEA published the *Australian Credentialled Diabetes Educators and Prescribing of Insulin and Glucose Lowering Agents - Scoping paper*. Whilst the document stipulated that ADEA did not endorse prescribing practices, it did state, ‘Some CDEs, such as a registered nurse or pharmacist, may through delegation or referral from an authorised medical practitioner accept secondary prescribing responsibilities ... ’ ([43], p.13). A subsequent document published in 2015, *Australian Credentialled Diabetes Educators and Prescribing of Insulin and Glucose Lowering Agents,* further detailed the actions required to progress ADEA’s ambition to extend the scope of practice of CDEs to include non-medical prescribing. It stated that, ‘The difference between the role of the nurse practitioner (diabetes) and future RN CDE with prescribing rights should be delineated’ ([43], p.5).

In 2015, the Role and Scope of Practice for Credentialled Diabetes Educators in Australia was updated. Unlike the preceding version this revision stated explicitly, ‘The current scope of practice of the CDE does not include prescribing or titrating of any medications, unless there is legislated change or endorsement of these functions by state and territory governments’ ([13], p.18). Legislation appears to be the most salient factor guiding the perceived roles and scopes of practice of diabetes educators and yet with the legislative changes affecting the different CDE eligible professions that have occurred over time, the role boundaries and scopes of practice of diabetes educators of different backgrounds have become arguably more ambiguous.

### Expansion of the Medicare benefits schedule era

Medicare is Australia’s publicly funded national health insurance system which has been in place since 1975. In its early years, Medicare benefits were almost exclusively accessible by the medical profession [44]. Throughout 1985–86, the Layton Inquiry was undertaken, which, in part, sought to determine whether the Medicare Scheme should be expanded to enable other, non-medical health services to access benefits for their services. Some 22 non-medical health professions made submissions, seeking inclusion in the Medicare Scheme. The Australian Medical Association opposed the expansion of the Medicare Scheme. The outcome was that Medicare benefits would remain as they were: available to the medical profession and optometry with very restricted benefits for dental services [45, 46].

Almost 20 years later, in 2004, podiatrists, dietitians, mental health nurses and dentists were included in the Medicare Benefits Schedule (MBS), as it came to be known. These professions could apply for a Medicare provider number and provide services attracting partial Medicare rebates to patients, for patients with a specific type of referral from a medical practitioner [47]. This was a significant event for the health professions concerned, as it enabled them to bulk bill or offer significantly subsidised services in the private sector. Subsequently, ADEA CDEs were included in the MBS [48, 49]. The MBS has since expanded further to include more non-medical health services [45, 46].

Around the time that CDEs were first included in the MBS, several professional associations approached ADEA seeking eligibility for credentialling. In 2006, ADEA reported, ‘A number of disciplines approached ADEA for eligibility for CDE® and the review has been conducted to assess eligibility against agreed criteria’ ([50], p. 8)*.* Pharmacists were added to the CDE eligibility list in 2007, podiatrists in 2008, exercise physiologists in 2012, direct entry midwives and physiotherapists in 2015. In 2016, ADEA reviewed the process and standards used to evaluate applications for CDE eligibility made by professional bodies [32]. The criteria used to determine the eligibility of professions, while referred to as ‘relevant and robust’, are not available to the wider public [31].

### The competency movement

The competency movement began in the 1990s when the commonwealth government sought to introduce a nationally consistent approach to the training and qualification of workers across a range of industries. Competency standards serve as a quality assurance measure, reflecting the appropriate application of sound knowledge and skills within a particular vocational context [51, 52]. In 1994, ADEA instigated the development of competencies for diabetes educators and in 1996, published *National Core Competencies for Diabetes Educators*. Subsequently a paper entitled, *The process of developing and validating national core competencies for diabetes educators,* was published in a peer-reviewed journal [51]. The authors define the field of diabetes education as *interdisciplinary*. ADEA’s *Core Competencies* document provided examples of clinical competencies, defined according to primary discipline: ‘ ... nurses: insulin dosage adjustment or correct injection technique; dietitians: diabetes dietary prescription; podiatrists: wound care’ ([23], p.3).

In 2005, the Productivity Commission released a research report, *Australia’s Health Workforce,* which examined the issues affecting Australia’s health care workforce. The report described factors inhibiting health workforce innovation such as entrenched custom and practice, limiting role flexibility and impeding the ability of the workforce to meet its full potential [3]. It was acknowledged that traditional health care roles and boundaries have their place, ensuring high quality patient care, however historical and rigid work practices can, ‘ … impede transferability of skills across professional boundaries; prevent appropriate recognition of prior learning; constrain the move to a more competency-based education and training system; and discourage the further development of multidisciplinary care approaches’ ([3], p.29).

Furthermore, it indicated that professional bodies often implement strategies such as setting entry criteria and developing codes of conduct, which are primarily designed to uphold standards of quality and safety. These strategies, however, may also be driven by the desire to protect the professional task domain and associated income [3]. In an appendix within ADEA’s *Role and Scope of Practice* [20] document, there is reference to the Productivity Commission’s 2005 Report:

The Productivity Commission Report is calling for professional boundaries and discipline specific practice to be broken down, for more interdisciplinary practice and for work place innovation. On the other hand, many disciplines and their governing or professional bodies are advocating recognition of advanced specialisations. In defining the role and scope of practice of the Credentialled Diabetes Educator, ADEA must be ready to embrace these possible changes ... ([20], p.18).

ADEA’s mentoring program was launched in 2008 [53]. A previous publication, *Credentialling of Diabetes Educators 2000,* stated that mentoring partnerships *may* be established between diabetes educators of different primary professions. In 2016, cross-discipline mentor partnerships were not just permissible, but were recognised as, ‘ ... a way to learn and experience new ideas in a two-way partnership’ ([31], p. 41).

In 2012, the National Prescribing Service (NPS) published the *Competencies Required to Prescribe Medicines* report. The report presented a competency framework for potential non-medical prescribers [54]. The *Health Professionals Prescribing Pathway Project Final Report* published in 2013 referred to the NPS competency framework and discussed non-medical prescribing practices which were already taking place by professions such as podiatry, nursing and dentistry. The report did not refer to competencies specific to particular health professions, but rather a more general discussion about the key qualities and skills which are indicative of competence in prescribing [55].

ADEA’s most recent *Role and Scope of Practice for Credentialled Diabetes Educators in Australia* [13] document contained comparisons between the scopes of practice of diabetes educators in Australia, America and Canada. In America there are two diabetes educator certification pathways: the National Certification Board of Diabetes Educators and the American Association of Diabetes Educators (AADE). The professionals eligible for AADE certification include registered nurses, registered dietitians, registered pharmacists, physicians and physician assistants. There is a three-tiered approach to certification, with each tier corresponding to different levels of competencies. Each of the three certification levels can be obtained by any of the eligible professions. The system employed by the Canadian Diabetes Educator Certification Board is similar to ADEA’s, whereby diabetes educators are bound by the competencies specific to their primary profession. At the conclusion of ADEA’s international comparison, it stated,

Currently there exists debate about the level of competency for each discipline undertaking accredited courses in diabetes education and management. Many members would like to see a level playing field and competency outcomes developed for each of these courses. ADEA will explore this issue with the education facilities that offer the diabetes education and management course ([13], p. 25).

## Discussion

This analysis illustrates significant evolution in the diabetes educator role since the establishment of ADEA, in response to the changing social climate and health care policy in Australia. Initially considered part of the nursing role in the hospital setting, the education of people with diabetes has become increasingly recognised as an interdisciplinary role. There have been some key influences or drivers which have moved the association toward a more inclusive interdisciplinary entity. Earlier documents implied that there were defined clinical roles within the diabetes educator workforce, which correlated with the varying primary disciplines. There was little evidence of role flexibility. Terms such as multi- and inter-disciplinary began to be used more frequently in ADEA documents in the early 2000s, with more recent documents elaborating the terms and referring to the breaking down of professional role boundaries.

This review indicates that there were three key drivers that have influenced the diabetes educator role and scope of practice. These three events or drivers occurred at the macro level. There were events which occurred at the meso or professional association level, which were less cogent in their influence of the interprofessional role boundaries in diabetes education. Examples of such meso level events include the publication of ADEA and other professional documents, the addition of further professional groups for CDE eligibility, ADEA process reviews and communication to the membership. Micro level or events which reflected the status of the interprofessional role boundaries appeared to be even less influential and included the credentialling of individual non-nurse diabetes educators.

The first macro-level influence on diabetes educator role boundaries was the rescheduling of insulin from schedule III to IV which came into effect in December 2000. This significantly altered the role and scope of practice for RN diabetes educators at the time. This legislative change appears to have influenced further changes in ADEA and the diabetes educator workforce, as evidenced by the change of wording, predominantly in the standards of practice documents, which, after 2000, appeared to place less emphasis on the RN CDE’s role in adjusting insulin. Since the rescheduling of insulin, the nursing profession has sought to change the legislation to enable RN CDEs to undertake prescribing and medication supply practices to enhance their capacity and autonomy as diabetes educators. ADEA acknowledged that advances in CDE practice to include prescribing would signal the need to clarify the delineation between RN CDEs and diabetes NPs. References to the clarification of other professions’ role boundaries were notably absent, presumably because it was perceived that only RNs would benefit from this expansion of CDE scope of practice. In New South Wales at least, legislation permits RN CDEs to issue a patient with a seven day supply of insulin that has been prescribed by a medical practitioner. This has reinforced role boundaries between RN and non-nurse diabetes educators.

The second key driver identified was the expansion of the MBS to include benefits for allied health providers. Diabetes educators were added to the MBS in 2004, stimulating changes to the composition of the diabetes educator workforce. The Government’s decision to permit access to MBS rebates to diabetes educators who were ADEA credentialled made it more appealing to become credentialled. Furthermore, the potential to access Medicare rebates for services prompted a number of non-ADEA eligible professions, such as pharmacists and exercise physiologists, to seek eligibility. This was evidenced by the fact that more professions applied to the ADEA to become eligible for credentialling from 2005, prompting ADEA to review its procedures for assessing applications from professional bodies. ADEA continue to utilise its own criteria to determine the eligibility of professions to achieve CDE status. While a number of professions have since been deemed eligible by the ADEA, the criteria utilised to evaluate a professional body’s application are not publically available. As such, the ADEA remains the gatekeeper, determining which professions are eligible for credentialling and therefore which professions can benefit from the MBS rebates for CDE services.

The third macro-level driver was the publication of the Productivity Commission Research Report [3]*.* The report was considered a major impetus for changing thinking around the way health services were planned and delivered [56]. It addressed various factors affecting the health care workforce’s productivity, highlighting the difficulty in determining the capacity of the workforce due to the emphasis on professions, rather than competencies. The report made a number of recommendations to improve the efficiency and productivity of the health care workforce, such as the cultivation of a supportive workplace where traditional role boundaries can be re-negotiated in favour of interdisciplinary practice. ADEA’s 2007 role and scope of practice document [20] made reference to this recommendation and the possibility that the diabetes educator workforce may need to embrace this innovative approach to health care provision. Prior to this publication, ADEA had used the term *interdisciplinary*, however, this was the earliest document included in the review that elaborated on it and referred breaking down role boundaries. ADEA documents published after 2007 placed less emphasis on the specific clinical skills and task domains correlating with the different primary disciplines. In their comparison with international systems and processes by which diabetes educators are credentialled in 2015, ADEA demonstrated an interest in pursuing a more competency-based focus, in line with recent health policy research.

This analysis demonstrates that the capacity of the health care workforce to evolve in response to macro level influences such as health care policy, modernizing changes and legislation may be hindered by the workforce itself. Resistance to changes to improve role flexibility may arise in the form of social processes and profession-based strategies to preserve traditional role boundaries and ways of working. These findings may be of relevance to other contexts where interprofessional role boundaries are ambiguous or contested, such as mental health.

## Conclusion

This analysis illustrates the gradual movement of the Australian diabetes educator workforce from a nursing dominant entity with an emphasis on interprofessional role boundaries to an interdisciplinary body in which role flexibility is encouraged. ADEA is striving to foster an interdisciplinary culture to strengthen and advance this faction of the health care workforce and has demonstrated interest in adopting contemporary approaches to the delivery of diabetes self-management education. However, this analysis also demonstrates that strategies to exclude non-nurse diabetes educators from practising to the same level as RN CDEs with regards to non-medical prescribing remain apparent.

## Additional files

Additional file 1: Documents included in analysis. (DOCX 21 kb)

Additional file 2: Quality indicators for documents included. (DOCX 15 kb)
