# Supplementary material for: Diabetes educator role boundaries in Australia: a documentary analysis
Source: J Foot Ankle Res. 2017 Jul 10;10:28. doi: 10.1186/s13047-017-0210-9 (PMC5504808; doi:10.1186/s13047-017-0210-9)
Supplement: Supplementary file 1 — Documents included in analysis. (DOCX 21 kb) [file 13047_2017_210_MOESM1_ESM.docx]

# Additional file 1

|  | ***Title*, Year** | **Author** | **URL (if available)** |
| --- | --- | --- | --- |
| Current ADEA* standards, position statements and clinical guidelines | *Role and Scope of Practice for Credentialled Diabetes Educators in Australia,* 2015  *The Role of Credentialled Diabetes Educators and Accredited Practising Dietitians in the Delivery of Diabetes Self Management and Nutrition Services for People with Diabetes,* 2015  *Constitution*  *National Standards for the Development and Quality Assessment of Services; Initiating Insulin Therapy in the Ambulatory Care Setting,* 2012 | ADEA  ADEA and Dietitians Association of Australia    ADEA  ADEA | <https://www.adea.com.au/wp-content/uploads/2009/10/Role-and-Scope-of-Practice-for-Credentialled-Diabetes-Educators-in-Australia-Final1.pdf>  <https://www.adea.com.au/wp-content/uploads/2009/10/Draft-CDE-APD-Role-Statement_Final.pdf>  <https://www.adea.com.au/wp-content/uploads/2015/07/150522_Constitution_FINAL.pdf>  <https://www.adea.com.au/wp-content/uploads/2009/10/Draft_Insulin_Standards_Version_1_2_Jan_2013_2_1425352580160.pdf> |
| Superseded ADEA standards, position statements and clinical guidelines | *National Guidelines for the Safe Practice of Diabetes Nurse Educators,* 1994  *National Standards of Practice for Diabetes Educators,* 1991  *National Standards of Practice for Diabetes Educators,* 2003  *National Core Competencies for Diabetes Educators,* 1996  *National Core Competencies for Diabetes Educators,* 2001  *National Core Competencies for Diabetes Educators,* 2008  *Joint Statement on the Role of Accredited Practising Dietitians and Diabetes Educators in the Delivery of Nutrition and Diabetes Self-Management Education Services for People with Diabetes,* 2005  *The Role of the Diabetes Educator in Australia,* 2001  *The Credentialled Diabetes Educator in Australia Role and Scope of Practice,* 2007  *The National Standards for Developing and assessing the Quality of Services: Initiating Insulin Therapy in Ambulatory Settings,* 2004 | ADEA  ADEA  ADEA  ADEA  ADEA  ADEA  ADEA and Dietitians Association of Australia  ADEA  ADEA  ADEA | Full references contained in reference list within the paper  <https://www.adea.com.au/wp-content/uploads/2013/08/NStof_practice_for_DEs.pdf>  <https://www.adea.com.au/wp-content/uploads/2013/08/Core_Comp_CDEs.pdf>  <https://www.adea.com.au/wp-content/uploads/2009/10/ADEA-DAA-position-statement-re-role-of-CDE-and-dietitians-endorsed-DAA-2009.pdf>  <https://www.adea.com.au/wp-content/uploads/2013/08/The_CDE_Role_and_scope.pdf> |
| ADEA Annual Reports | *Annual Report,* 2006  *Annual Report,* 2007  *Annual Report,*2008  *Annual Report,*2009  *Annual Report,*2010  *Annual Report,*2012  *Annual Report,*2014  *Annual Report,*2015  *Annual Report,*2016 | All Annual Reports authored by ADEA | <https://www.adea.com.au/wp-content/uploads/2013/08/ADEA_Annual_Report_2005-06.pdf>  <https://www.adea.com.au/wp-content/uploads/2013/08/ADEA_Annual_Report_2006-07.pdf>  <https://www.adea.com.au/wp-content/uploads/2013/08/ADEA_Annual_Report_2007-08.pdf>  <https://www.adea.com.au/wp-content/uploads/2013/08/ADEA_Annual_Report_2008-09.pdf>  <https://www.adea.com.au/wp-content/uploads/2013/08/ADEA_Annual_Report_2009-2010_for_web.pdf>  <https://www.adea.com.au/wp-content/uploads/2013/08/ADEA_AnnRep_2011-12_13Feb13.pdf>  <https://www.adea.com.au/wp-content/uploads/2009/10/annual-report-2014-final-version-low.pdf>  <https://www.adea.com.au/wp-content/uploads/2016/09/Annual-Report-2015-final-web-12082015.pdf>  <https://www.adea.com.au/wp-content/uploads/2016/09/annual-report-2015-16-Final-Web-updated-very-low.pdf> |
| ADEA meeting minutes | *31st Annual General Meeting Australian Diabetes Educators Association Gold Coast Convention Centre*, 30 August 2012  *Special Resolution Meeting Minutes (Constitutional Changes),* 2016 | ADEA  ADEA | <https://www.adea.com.au/wp-content/uploads/2013/08/2012_AGM_Minutes.pdf>  <https://www.adea.com.au/wp-content/uploads/2014/05/160418__ADEA-General-Meeting_Minutes_FINAL1.pdf> |
| ADEA Submissions | - [*Proposed expanded endorsement for scheduled medicines. Draft Registration standard for endorsement of registered nurses and/or registered midwives to supply and administer scheduled medicines under protocol*](https://www.adea.com.au/wp-content/uploads/2013/12/ADEA-Response_NMBA_FINAL.pdf)*.* Submission to the Nursing and Midwifery Board of Australia, 2010 | ADEA | <https://www.adea.com.au/wp-content/uploads/2013/12/ADEA-Response_NMBA_FINAL.pdf> |
| ADEA Project / scoping / information documents | *Australian Credentialled Diabetes Educators & Prescribing of Insulin & Glucose Lowering Agents,* 2015 | ADEA | <https://www.adea.com.au/wp-content/uploads/2013/08/150409_Australian-CDEs-and-Prescribing-of-Insulin-and-glucose-lowering-agents_FINAL-APPROVED.pdf> |
| Member communication | *Working for All Members – Communiqué,* 2016  *Insulin RN CDE Resolution Process, 2013* | ADEA  ADEA | <https://www.adea.com.au/members/working-for-all-members/>  <https://www.adea.com.au/wp-content/uploads/2013/10/Insulin-RN-CDE-resolution-progress_v2.pdf> |
| Project reports, gazettes, legislation | *Gazette,* 2000 (GN12 p. 746-747)  *Gazette, Legislation,* 2001 (190)  *Medicare Benefits Schedule Book,* 2004  *Medicare Benefits Schedule Book,*2005  *Australia’s Health Workforce*, 2005  *NPS: Better choices, Better health. Competencies required to prescribe medicines: putting quality use of medicines into practice, 2012*  *Overview of Commonwealth involvement in funding dental care,* 2008  *Health Professionals Prescribing Pathway (HPPP) Project Final Report,* 2013 | Commonwealth of Australia  NSW** Government  Australian Government Department of Health and Ageing  Productivity Commission  National Prescribing Service Limited  Biggs, A.  Health Workforce Australia | <file:///D:/Users/Olivia/Downloads/2000GN12.pdf>  <http://gazette.legislation.nsw.gov.au/so/download.w3p?id=Gaz_Gazette%20Split%202001_2001-190.pdf>  <http://www.mbsonline.gov.au/internet/mbsonline/publishing.nsf/Content/MBSOnline-2000>  <http://www.mbsonline.gov.au/internet/mbsonline/publishing.nsf/Content/MBSOnline-2000>  <http://www.pc.gov.au/inquiries/completed/health-workforce/report/healthworkforce.pdf>  <https://cdn0.scrvt.com/08ab3606b0b7a8ea53fd0b40b1c44f86/ab0cc7f2a28cc4a1/152fb1f49b28/Prescribing_Competencies_Framework.pdf>  <http://www.aph.gov.au/About_Parliament/Parliamentary_Departments/Parliamentary_Library/pubs/rp/rp0809/09rp01>  <http://www.healthinfonet.ecu.edu.au/key-resources/bibliography/?lid=26503> |
| Peer reviewed literature | *Non-medical prescribing in Australasia and the UK: the case of podiatry,* 2010 | Borthwick, A., Short, A., Nancarrow, S. & Boyce, R. | <https://jfootankleres.biomedcentral.com/articles/10.1186/1757-1146-3-1>  Full reference contained in reference list within the paper |
| Emails to clarify details | Email communication, 30/08/2016  Email communication, 03/10/2016  Email communication, 09/10,2017 | ADEA employee #1  ADEA employee #2  ADEA employee #1 |  |
| Peer reviewed literature | *The process of developing and validating national core competencies for diabetes educators,* 1996 | Colagiuri, R. & Ritchie, J. | <http://www.publish.csiro.au/AH/AH960029> |
| Written by diabetes educators about diabetes education practice | *Diabetes education in Australia,* 1984  *All about diabetes educators -- a guide for general practitioners,* 2005  *Nursing roles in initiating and adjusting insulin,* 2010  *Diabetes Educators Get Item Numbers,* 2004 | Cusworth, L.  Alford, J.  Giles, J.  Australian Nursing & Midwifery Federation | Full references contained in reference list within the paper |

*ADEA = Australian Diabetes Educators Association

**NSW= New South Wales
