# Supplementary material for: Diabetes educator role boundaries in Australia: a documentary analysis
Source: J Foot Ankle Res. 2017 Jul 10;10:28. doi: 10.1186/s13047-017-0210-9 (PMC5504808; doi:10.1186/s13047-017-0210-9)
Supplement: Supplementary file 2 — Quality indicators for documents included. (DOCX 15 kb) [file 13047_2017_210_MOESM2_ESM.docx]

# Additional file 2

| **Source** | **Literature type** | **Description** | **Number of records** | **Validity Indicator** | | | |
| --- | --- | --- | --- | --- | --- | --- | --- |
|  |  |  |  | **Authenticity** | **Credibility** | **Representativeness** | **Meaning** |
| ADEA* (retrieved from website) | Grey literature | Standards of practice ,position statements and clinical guidelines (current) | 4 | Produced by ADEA, published on website | Authored by ADEA | Documents represented ADEA’s position. Were available on ADEA website | Purpose of document stated in most cases |
| ADEA (retrieved via university library) | Grey | Standards of practice, position statements and clinical guidelines (superseded) | 10 | Produced by ADEA; were published and held at university libraries or the National Library of Australia (NLA) | Authored by ADEA | Documents represented ADEA’s position. Were held at libraries | Purpose of document stated in most cases |
| ADEA (retrieved from website) | Grey | Annual Reports | 9 | Produced by ADEA, published on website | Authored by ADEA | Annual reports provided details of key activities undertaken by ADEA over previous financial year | Meaning was clear due to nature of reports |
| ADEA (retrieved from website) | Grey | ADEA meeting minutes | 2 | Produced by ADEA, published on website | Authored by ADEA | A record of proceedings of ADEA AGM | Meaning was clear due to nature of document |
| ADEA (retrieved from website) | Grey | Submissions | 1 | Produced by ADEA, published on website | Authored by ADEA | Submission based on evidence gathered by ADEA and endorsed by CEO | Intention of submission was stated |
| ADEA (retrieved from website) | Grey | Project / scoping / information documents | 1 | Produced by ADEA, published on website | Authored by ADEA | Documents were developed by ADEA and available of their website | Purposes of documents were stated |
| ADEA (retrieved from website) | Grey | Member communication | 2 | Produced by ADEA, published on website | Authored by ADEA | ADEA newsletters and communiqués distributed to membership | Meaning was clear: these documents provided the member with targeted information |
| Government or Government agency documents (retrieved online) | Grey | Project reports, gazettes, legislation | 8 | Produced by government agencies, with relevant logos or endorsements evident | Government agencies are considered credible sources | Government documents are considered to be of high authority and are widely available online | Purposes of documents were stated most cases |
| Evidence to clarify specific facts | Peer reviewed | Published paper about the history of the podiatry profession | 1 | Published in a peer-reviewed journal | Peer review process enhances the credibility of the paper | Published in a journal and is available upon searching | Purpose of paper was stated |
| Email | Personal communication | Emails to clarify facts which are contracted or unaddressed in the documents retrieved | 3 | Only those deemed to be key informants or experts were consulted to clarify facts | Key informants demonstrated significant knowledge of the diabetes educator workforce and ADEA | Information was obtained via email, which can be kept for a designated period of time and made available as required | Personal communication was sought to clarify particular key points |
| Databases (CINAHL and Medline) | Peer reviewed | Published documents | 1 | Published in a peer-reviewed journal | The authors’ credentials included in paper. Both considered highly credible | Papers were accessible | Purpose of paper was stated |
| Database (CINAHL and Medline) | Opinion pieces | Written by diabetes educators about diabetes education practice | 4 | Published in journals | Authors were either credentialled diabetes educators or affiliated with ADEA | Documents were accessible | Purpose of piece was stated |

*ADEA = Australian Diabetes Educators Association: the national association for diabetes educators
